# Use, type and duration of cardiopulmonary bypass are related to acute kidney injury occurrence and urinary NGAL concentrations

**DOI:** 10.1186/cc12360

**Published:** 2013-03-19

**Authors:** T García Rodríguez San Miguel

**Affiliations:** 1 Hospital Santa Creu i Sant Pau, Barcelona, Spain

## Introduction

To analyze whether variables related to cardiopulmonary bypass (CPB) influence acute kidney injury (AKI) occurrence and urinary neutrophil gelatinase-associated lipocalin (uNGAL) in cardiac surgery patients.

## Methods

A total of 274 adult cardiac surgery patients were consecutively included from February to December 2011. Exclusion criteria were absence of diuresis due to end-stage renal disease or chronic renal failure and cardiac catheterism with i.v. contrast in the week before surgery. CPB, when performed, was used as standard CPB (SCPB) or MiniCPB. We obtained four serial blood and urine samples, immediately before (PRE) and after (POST) surgery, and 1 day (1d) and 2 days (2d) after surgery. uNGAL was measured by Architect 6200 (Abbott Diagnostics). AKIN criteria were used to diagnose AKI. The study was approved by the local ethics committee and all patients gave informed consent.

## Results

One hundred and eighty-one patients (66.1%) were men; mean age was 68.2 ± 12.2 years. ICU and hospital stays were 6.7 ± 8.1 and 15.7 ± 13.9 days, respectively. Twenty-eight-day mortality was 2.9%. Eighty-six patients (31.4%) were diagnosed with AKI within 48 hours after surgery. In total, 219 patients required CPB (195 SCPB, 24 MiniCPB) and 55 did not (no-CPB). Seven no-CPB patients (12.7%) developed AKI and their median uNGAL POST was 330 (42.6 to 489.9) μg/l compared with 13.6 (6.9 to 38.3) μg/l in the 48 patients without AKI (*P *0.0001). Of the 195 patients undergoing SCPB, 76 (38.9%) developed AKI and 119 did not; POST uNGAL was 204 (34.8 to 575.7) μg/l and 44.5 (13.2 to 175.8) μg/l (*P *0.0001), respectively. In the 24 patients under MiniCPB, POST uNGAL was 113 (58.8 to 211.8) μg/l in those (three patients, 12.5%) developing AKI and 19.1 (9.2 to 41.8) μg/l in those without AKI (*P *= 0.01). Aortic clamp time (*r *= 0.31, *P *0.0001) and cardiopulmonary bypass time (*r *= 0.30, *P *0.0001) correlated with POST uNGAL concentrations. Incidence of AKI in patients without CPB or with MiniCPB was identical and significantly lower (*P *0.0001) than in SCPB patients.

## Conclusion

The incidence of AKI was higher in patients undergoing SCPB. Aortic clamp and cardiopulmonary bypass time correlated with the POST uNGAL concentration. The higher values found in patients without AKI undergoing SCPB suggest that a subclinical AKI, only detectable by uNGAL, could exist in this group.

