# Correction: The use, type and duration of cardiopulmonary bypass are related to acute kidney injury occurrence and urinary NGAL concentrations

**DOI:** 10.1186/cc12605

**Published:** 2013-04-19

**Authors:** T García, AJ Betbesé, M García, V Baños, R Bonet, J Ordonez-Llanos

**Affiliations:** 1 Intensive Care, Hospital de la Santa Creu i Sant Pau, Barcelone, Spain; 2 Anethesiology Department, Hospital de la Santa Creu i Sant Pau, Barcelone, Spain; 3 Biochemistry Department, Hospital de la Santa Creu i Sant Pau, Barcelone, Spain

## Correction

After the publication of this abstract [1], we found that not all authors were listed. A full author list is provided here. We regret any inconvenience this error may have caused.
